# JMJD3 suppresses stem cell-like characteristics in breast cancer cells by downregulation of Oct4 independently of its demethylase activity

**DOI:** 10.18632/oncotarget.15747

**Published:** 2017-02-26

**Authors:** Jing Xun, Dekun Wang, Long Shen, Junbo Gong, Ruifang Gao, Lingfang Du, Antao Chang, Xiangrong Song, Rong Xiang, Xiaoyue Tan

**Affiliations:** ^1^ College of Medicine, State Key Laboratory of Medicinal Chemical Biology, Nankai University, Tianjin 300071, China; ^2^ Tianjin Key Laboratory of Modern Drug Delivery and High Efficiency in Tianjin University, School of Chemical Engineering and Technology, Tianjin University, Tianjin 300072, China; ^3^ State Key Laboratory of Biotherapy and Cancer Center, West China Hospital, Sichuan University, Chengdu 610041, China

**Keywords:** JMJD3, Oct4, cancer stem cell-like characteristic, PHF20, paricalcitol

## Abstract

Epigenetic regulator JMJD3 plays an important role in both tumor progression and somatic cell reprogramming. Here, we explored the effect of JMJD3 on the stem cell-like characteristics of breast cancer and its underlying mechanism involving stemness-related transcription factor Oct4. Our data revealed that, in breast cancer cells lines and an orthotopic xenograph mouse model of breast cancer, ectopic overexpression of JMJD3 suppressed stem cell-like characteristics of breast cancer cells, whereas knockdown of JMJD3 promoted these characteristics. Oct4 mediated the suppressive effects of JMJD3 on the stemness of breast cancer cells. The inhibitory effect of JMJD3 on Oct4 was independent of demethylase activity, but mediated via degradation of PHF20. Furthermore, we applied an agonist of the vitamin D receptor, paricalcitol, and found that it induced JMJD3 in breast cancer cells. Our data showed that administration of paricalcitol suppressed stem cell-like characteristics and Oct4 expression. Taken together, JMJD3 inhibits the stem cell-like characteristics in breast cancer by suppression of stemness factor Oct4 in a PHF20-dependent manner. Administration of paricalcitol leads to upregulation of JMJD3 that suppresses Oct4 expression and the stem cell-like characteristics in breast cancer.

## INTRODUCTION

Post-translational modifications of histone tails play critical roles in development, differentiation, and cancer [1]. Transcriptional activation and repression maintain a dynamic balance to achieve fine-tuned gene regulation under the control of various factors associated with methylation and acetylation [2, 3, 4]. Jumonji domain-containing-3 (JMJD3), one of two histone H3K27me3 demethylases, has been reported to participate in the regulation of tumorigenesis [5, 6]. However, there are conflicting studies about the effects of JMJD3 on tumors. In human diploid fibroblasts, JMJD3 serves as a tumor suppressor by binding to and activating the INK4A-ARF locus [7, 8], while in T cell acute lymphoblastic leukemia, it functions as an oncogene required for leukemia initiation and maintenance [9, 10]. Another interesting aspect is that accumulating evidences support functions of JMJD3 independent of its demethylase activity. Zhao et al. reported that JMJD3 inhibits the expression of the transcription factor Oct4 during reprogramming of fibroblasts through accelerating PHF20 ubiquitination in a demethylase-independent manner [11]. Obviously, the biology and pathological effects of JMJD3 are complex and extend beyond its demethylase activity. Further exploration of the role of JMJD3 in tumorigenesis and its underlying mechanism is required to find new therapeutic targets for cancer treatment.

Oct4 (Octamer-binding transcription factor 4) is considered as a key factor in maintaining and inducing pluripotency [12]. It has been more well known as one of the “Yamanaka factors” for the generation of induced pluripotent stem cells (iPSCs) [13]. Oct4 plays a key role in somatic reprogramming during tumorigenesis [14]. Knockdown of Oct4 in murine and human cell lines leads to suppression of tumor growth and decreases the cancer stem cells (CSCs)-like populations [15, 16]. An exclusive capacity for tumorigenesis in secondary recipients is the key property of CSCs, in addition to other critical properties they share with embryonic stem cells, such as unlimited self-renewal, a multilineage differentiation potential, and maintenance of the stemness state [17]. Therefore, Oct4 was tightly correlated with the processes of tumorigenesis. Here, we studied the effect of JMJD3 via regulation of Oct4 on tumorigenesis, and its underlying mechanism in breast cancer.

Several studies indicated that the active vitamin D metabolite 1a,25-dihydroxyvitamin D_3_ [1,25(OH)_2_D_3_] has a regulatory action in the expression of genes encoding histone demethylases of JMJD3 and lysine-specific demethylase families [18]. Typically, the action of 1,25(OH)_2_D_3_ implies activation of the vitamin D receptor (VDR) that binds specific DNA sequences within its target genes and modulates their transcription rate [19]. In colon cancer, 1,25(OH)_2_D_3_ activates the JMJD3 gene promoter and increases the level of JMJD3 in human cancer cells, which is strictly dependent on VDR expression [20]. In this study, a synthetic vitamin D analog, paricalcitol was used to induce the expression of JMJD3 in cancer cells to explore the mechanism underlying the effect of JMJD3 *in vivo* and evaluate its utility for clinical tumor therapy.

## RESULTS

### JMJD3 suppresses stem cell-like characteristics in breast cancer cells

**Figure 1:**
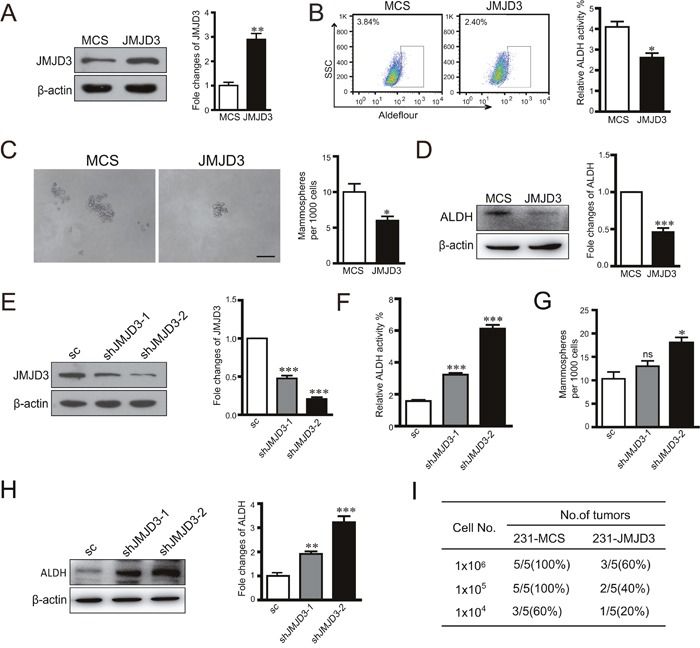
Overexpression of JMJD3 suppresses while silencing down of JMJD3 promotes stem cell-like characteristics in MDA-MB-231 cells We established stable JMJD3-overexpressing and control MDA-MB-231 cell lines. **(A)** Efficiency of JMJD3 overexpression detected by western blotting. The quantification of relative protein level is shown at the right panel. **(B)** Flow cytometric analysis of ALDH activity in JMJD3-overexpressing and control cell lines. Statistical results are shown in the right panel. **(C)** Representative images of sphere formation assays. Statistical results are shown in the right panel. Scale bar = 100 μm. **(D)** ALDH expression was detected by western blotting in stable JMJD3-overexpressing and control MDA-MB-231 cells. The quantification of relative ALDH level is shown at the right panel. Then, we established stable JMJD3 knockdown and scramble control MDA-MB-231 cell lines. **(E)** Efficiency of JMJD3 knockdown detected by western blotting. The statistical result is shown at the right penal. **(F** and **G)** Results of ALDH activity and sphere formation assays. Data are from three independent experiments and are shown as the mean ± S.E.M.*P<0.05 and ***P<0.001. **(H)** ALDH expression was detected by western blotting in stable JMJD3 knockdown and scramble control MDA-MB-231 cells. The statistical results are shown at the right penal. **(I)** Limited dilutions of stable JMJD3-overexpressing and control MDA-MB-231 cells were subcutaneously injected into the fat pads of female BALB/C nude mice (n=5). Tumors were monitored every 2 days by manual palpation for 2 weeks. The tumorigenic capacity is shown in the table.

We first evaluated the effect of histone H3K27me3 demethylase JMJD3 on the stem cell-like characteristics of breast cancer cells by stable overexpression or knockdown of JMJD3 in a cultured breast cancer cell lines, MDA-MB-231. Stem cell-like characteristics were measured by flow cytometric analysis of ALDH activity, a sphere formation assay, and western blotting of ALDH *in vitro*. The results showed significant suppression of ALDH expression, ALDH activity, and sphere formation by overexpression of JMJD3 (Figure 1A–1D). Accordingly, knockdown of JMJD3 upregulated ALDH activity, expression of ALDH, and sphere formation of MDA-MB-231 cells (Figure 1E–1H). Determination of tumorigenic potential revealed that overexpression of JMJD3 in MDA-MB-231 cells suppressed the tumorigenic potential compared with the control *in vivo* (Figure 1I).

### JMJD3 inhibits expression of Oct4 and leads to suppression of the stem cell-like characteristics in breast cancer cells

**Figure 2:**
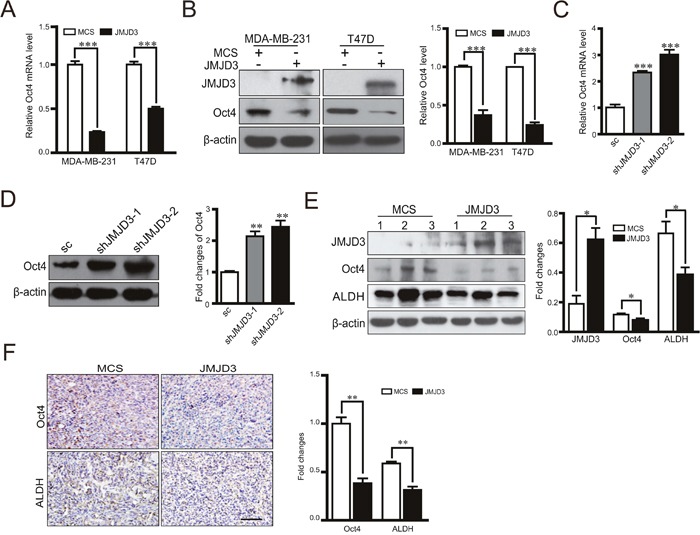
Overexpression of JMJD3 suppresses while silencing down of JMJD3 promotes Oct4 expression *in vitro* and *in vivo* We established JMJD3-overexpressing and control MDA-MB-231 or T47D cell lines. **(A)** mRNA expression of Oct4 in both cell lines detected by real-time PCR. **(B)** Expression level of Oct4 protein detected by western blotting. The result of quantification is shown in the right penal. Then, we established stable JMJD3 knockdown and scramble control MDA-MB-231 cell lines. **(C)** mRNA expression of Oct4 in stable cell lines detected by real-time PCR. **(D)** Expressional level of Oct4 protein detected by western blotting. The result of quantification is shown in the right penal. Data are shown as the mean ± S.E.M. of three independent experiments. *** P<0.001. Xenograft tumor models of breast cancer were established as described in Figure 1 using stable JMJD3-overexpressing and control MDA-MB-231 cell lines (n=5). **(E)** Representative western blots using antibodies against JMJD3, Oct4 and ALDH. Samples were homogenates of tumor tissues from various groups. Statistical results are shown in the right panel. Data are shown as the mean ± S.E.M.*P<0.05. **(F)** Representative images of immunohistochemical staining with antibodies against Oct4 and ALDH in tumor tissues. The right panel shows the statistical results. Scale bar =100 μm.

**Figure 3:**
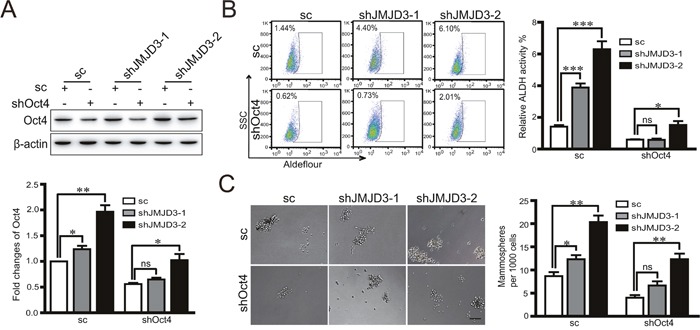
Oct4 rescues the promotion of stem cell-like characteristics induced by knockdown of JMJD3 Stable JMJD3 knockdown or scramble control MDA-MB-231 cells were transiently transfected with Oct4 shRNA or control plasmid. **(A)** Oct4 expression was detected by western blotting (top) and the bottom panel shows the statistical result. **(B)** Flow cytometric analysis of ALDH activity in JMJD3 knockdown and control cell lines. Statistical results are shown in the right panel. **P<0.01. **(C)** Representative images of the sphere formation assay. Statistical results are shown in the right panel. Scale bar = 100 μm. Data are shown as the mean ± S.E.M. of three independent experiments. ** P<0.01.

Considering the inhibitory effect of JMJD3 on the stem cell-like characteristics and critical role of Oct4 in tumorigenicity, we next tested whether JMJD3 affected the stemness-related transcription factor Oct4. Our data showed that overexpression of JMJD3 in MDA-MB-231 and T47D cells inhibited expression of Oct4 at both mRNA and protein levels (Figure 2A, 2B). Accordingly, knockdown of JMJD3 upregulated Oct4 expression (Figure 2C, 2D). Measurement of the Oct4 level in tumor tissue by western blotting and immunohistochemistry showed that Oct4 expression was significantly suppressed in the mouse model of breast cancer using stable JMJD3-overexpressing cells compared with the control, which was accompanied by a lower expression level of ALDH (Figure 2E, 2F). Furthermore, we explored the role of Oct4 in the effect of JMJD3 on the stem cell-like characteristics in breast cancer cells. The results showed that knockdown of Oct4 rescued the boost of ALDH activity and capacity of sphere formation caused by silencing-down of JMJD3 on (Figure 3A-3C). Taken together, it suggested that JMJD3 played an inhibitory role in Oct4 expression, and thereby led to its regulatory effect on the stem cell-like characteristics of breast cancer cells.

### Inhibitory effects of JMJD3 on Oct4 are independent of its demethylase activity in breast cancer cells

**Figure 4:**
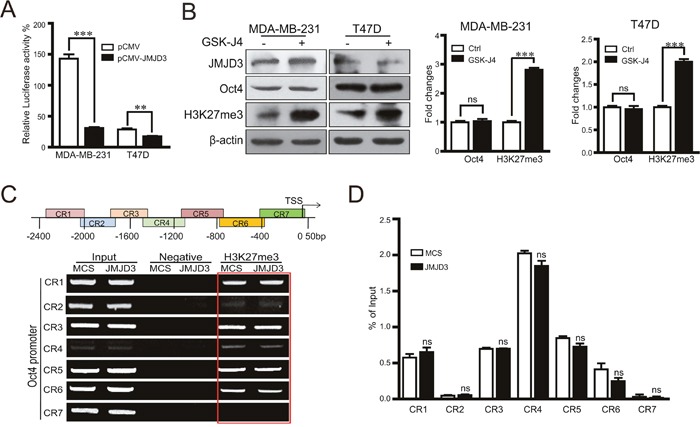
Effect of JMJD3 on the expression of Oct4 independently of its demethylase activity MDA-MB-231 and T47D cells were transfected with JMJD3 overexpression or control plasmid. **(A)** Results of dual-luciferase reporter assay detecting the promoter activity of Oct4. Data are shown as the mean ± S.E.M. of three independent experiments. ** P<0.01. **(B)** MDA-MB-231 and T47D cells were treated with 10 μM GSK-J4 for 48 h. Western blot analysis was performed using antibodies against JMJD3, Oct4 or H3K27me3. Statistical results are shown in the right panel. **(C** and **D)** ChIP-PCR analysis and statistical results of H3K27me3 marks on the Oct4 promoter in JMJD3-overexpressing and control MDA-MB-231 cells. Mouse IgG was used as the negative control with the input as the loading control.

To determine the mechanism underlying the inhibitory effect of JMJD3 on Oct4 in breast cancer cells, we first compared the activity of the Oct4 promoter in stable JMJD3-overexpressing and control cells by a dual luciferase assay. Our data showed that upregulation of JMJD3 suppressed the activity of the Oct4 promoter in both MDA-MB-231 and T47D cells (Figure 4A). Administration of the JMJD3 inhibitor GSK-J4 did not upregulate Oct4 expression, although the level of trimethylation of H3K27 was increased significantly (Figure 4B). These results suggest that the effect of JMJD3 on Oct4 is independent of its demethylase activity. Consistently, the ChIP assay did not identify increased binding upstream of the Oct4 promoter in stable JMJD3-overexpressing MDA-MB-231 cells compared with the control (Figure 4C, 4D).

### Inhibitory effects of JMJD3 on Oct4 depend on degradation of PHF20

**Figure 5:**
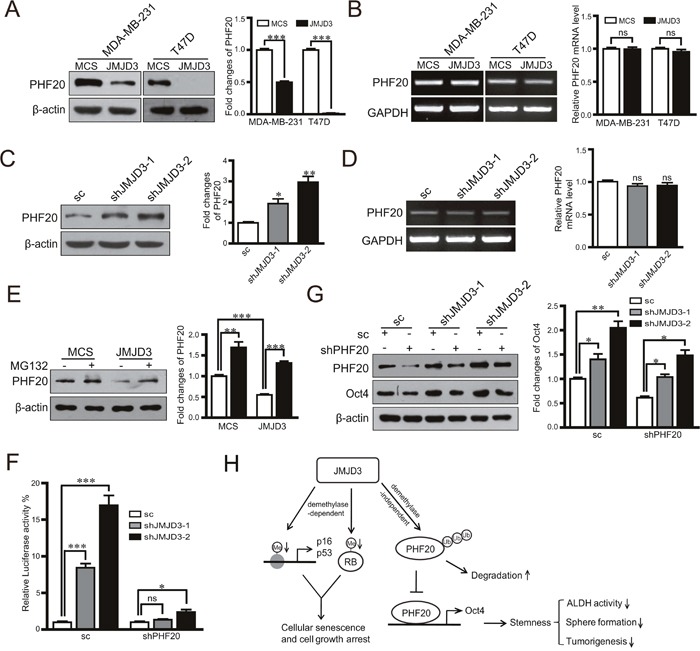
PHF20 mediates the regulatory effect of JMJD3 on the expression of Oct4 PHF20 expression at protein and mRNA levels detected by western blotting and RT-PCR in stable JMJD3-overexpressing and control MDA-MB-231/T47D cell lines **(A** and **B)**. The right penal is the statistical result respectively. PHF20 expression at protein and mRNA levels detected by western blotting and RT-PCR in stable JMJD3 knockdown and scramble control MDA-MB-231 cells **(C** and **D)**. The results of quantification are shown on the right panel. Stable JMJD3-overexpressing and control MDA-MB-231 cells were treated with 10 μM MG132 for 48 h. **(E)** PHF20 expression detected by western blotting and the quantification of protein level is shown on the right. PHF20 shRNA or the scrambled control were transfected into stable JMJD3 knockdown or scramble control MDA-MB-231 cells. **(F)** Results of dual-luciferase reporter assay detecting the promoter activity of Oct4. Data are shown as the mean ± S.E.M of three independent experiments. *P<0.05, ***P<0.001. **(G)** Western blot assay detecting the expression of PHF20 and Oct4. The right penal shows the statistical result of Oct4. **(H)** Schematic diagram shows the mechanism on the role of JMJD3 in regulation of stemness of breast cancer.

It was reported that inhibition of Oct4 by JMJD3 is a major rate-limiting step during the induction of iPSCs, and degradation of PHF20 through JMJD3 leads to inhibition of Oct4. Whether similar regulation exists during the acquirement of stem cell-like characteristics in cancer cells is still unknown. Our data showed that upregulation or knockdown of JMJD3 in breast cancer cells decreased or increased the protein level of PHF20, respectively, but did not change the mRNA expression level of PHF20 (Figure 5A-5D). To validate whether protein degradation accounts for the inhibition of PHF20 by JMJD3, we administered the proteasome inhibitor MG132 to JMJD3-overexpressing and control MDA-MB-231 cells. The results showed that inhibition of proteasomes abolished the inhibition of PHF20 induced by JMJD3 overexpression (Figure 5E). A dual-luciferase assay showed that knockdown of JMJD3 increased the activity of the Oct4 promoter, and the effect was significantly abolished by further knockdown of PHF20 (Figure 5F). Results of western blotting also showed that induction of Oct4 was suppressed by shRNA-mediated downregulation of PHF20 (Figure 5G). Taken together, our data suggest that inhibition of PHF20 by an increase in degradation mediates the effect of JMJD3 on Oct4 expression in breast cancer cells.

### VDR agonist paricalcitol induces JMJD3 as well as suppresses Oct4 and stem cell-like characteristics in breast cancer cells

**Figure 6:**
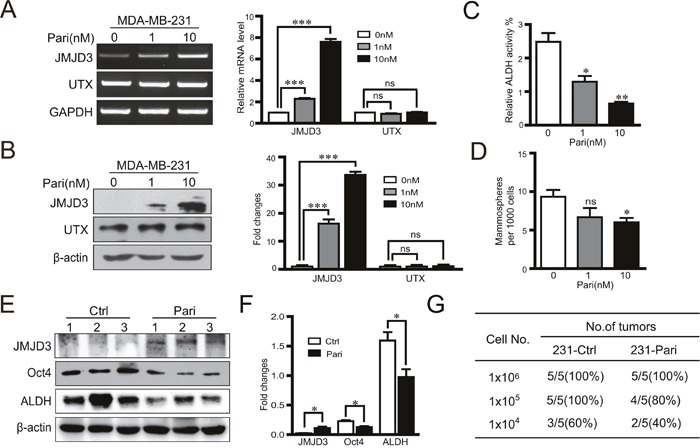
Vitamin D analogue paricalcitol induces JMJD3 and suppresses Oct4 and stem cell-like characteristics in MDA-MB-231 cells *in vitro* MDA-MB-231 cells were treated with 0, 1 or 10 nM paricalcitol for 48 h. **(A)** mRNA expression (left) and the statistical result (right) of JMJD3 and UTX detected by RT-PCR. **(B)** Expression of JMJD3 and UTX detected by western blotting and the quantification results are presented on the right. **(C** and **D)** Results of ALDH activity measurements by flow cytometry and sphere formation assays of MDA-MB-231 cells treated with paricalcitol for 7 days. Data are shown as the mean ± S.E.M of three independent experiments. *P<0.05, **P<0.01. A nude mouse model of breast cancer was established with MDA-MB-231 cells. Paricalcitol or the vehicle control were administrated via intraperitoneal injection at a dose of 0.3 μg/kg body weight once every 2 days from the 3 days before tumor cell implantation (n=5). Tumor samples were obtained at 8 weeks after injection for the following analysis. **(E** and **F)** Representative western blots of JMJD3, Oct4 and ALDH in primary tumor tissues. Statistical results are shown in the right panel. Data are shown as the mean ± S.E.M.*P<0.05, **P<0.01. **(G)** A limited dilution assay was performed *in vivo* with MDA-MB-231 cells. An orthotopic mouse model was established according to method described in Figure 1. The tumorigenic capacity is shown in the table.

To explore the potential for clinical translation, we administered a vitamin D analogue, VDR agonist paricalcitol, to cultured breast cancer cells and a mouse model of breast cancer. Our data showed that paricalcitol increased JMJD3 expression, but not UTX expression, in a dose-dependent manner at both mRNA and protein levels (Figure 6A, 6B). Moreover, paricalcitol significantly suppressed ALDH activity and sphere formation of cultured MDA-MB-231 cells (Figure 6C, 6D). In the orthotopic xenograph mouse model of breast cancer using MDA-MB-231 cells, expression of JMJD3 was increased in the tumor tissue while expression of Oct4 and ALDH was downregulated in the paricalcitol treatment group compared with the vehicle control (Figure 6E, 6F). Furthermore, administration of paricalcitol significantly suppressed the tumorigenic potential compared with the control (Figure 6G).

### JMJD3 is required for the inhibitory effects of paricalcitol on the stem cell-like properties in MDA-MB-231 cells

**Figure 7:**
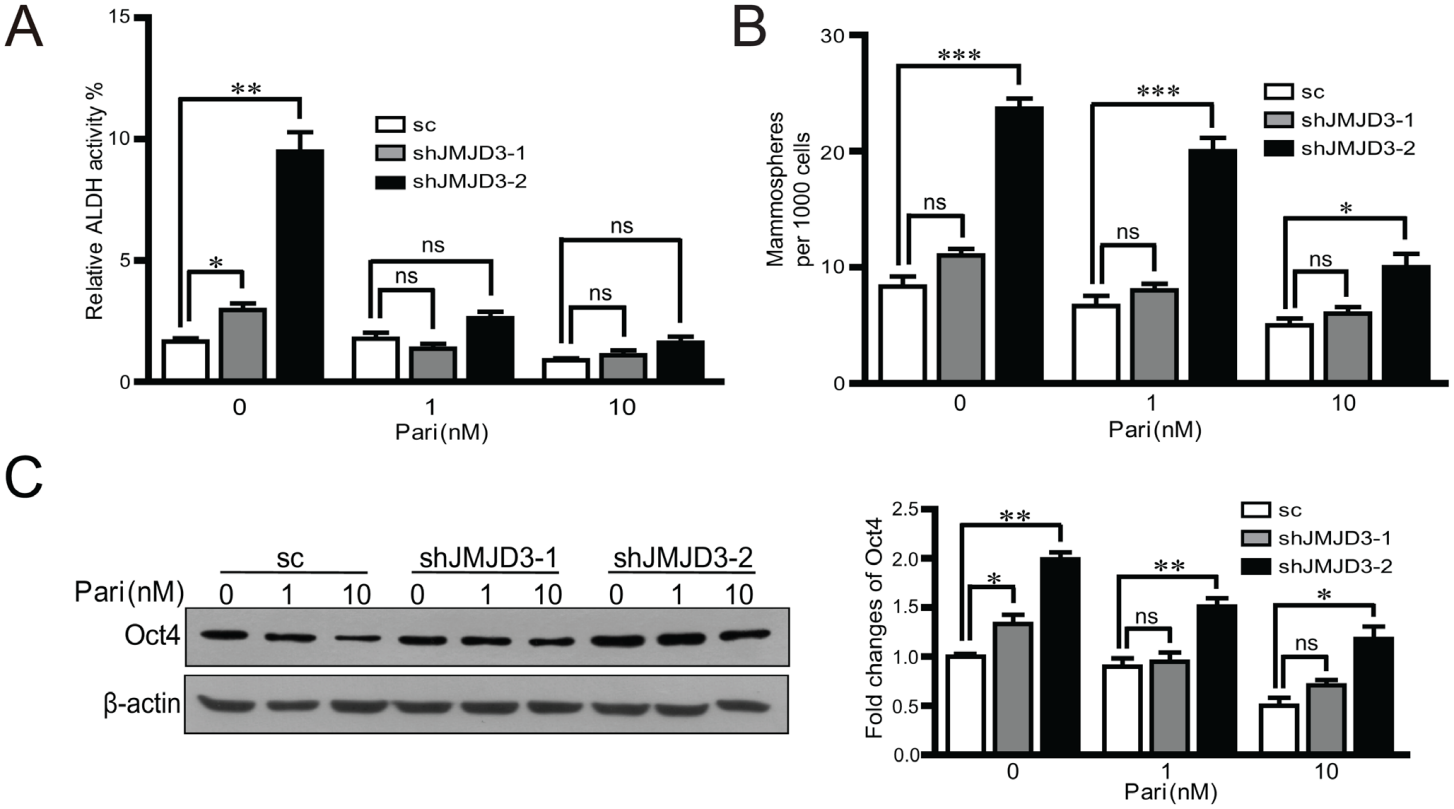
JMJD3 is required for the effects of paricalcitol on the stem cell-like properties in MDA-MB-231 cells Stable knockdown of JMJD3 or scramble control MDA-MB-231 cells were treated with 0, 1 or 10 nM paricalcitol for 48 h. **(A)** The analysis of ALDH activity by flow cytometry. **(B)** Result of sphere formation assay of stable JMJD3-knocking down and control MDA-MB-231 cells treated with paricalcitol for 7 days. Data are from three independent experiments and are shown as the mean ± S.E.M.*P<0.05 and ***P<0.001. **(C)** Expression of Oct4 detected by western blotting and the quantification results are presented in the right penal.

Further, we investigated whether JMJD3 was required for the inhibition of paricalcitol on the stem cell-like characteristics *in vitro*. The results showed that knockdown of JMJD3 rescued the inhibitory effect of paricalcitol on ALDH activity and capacity of sphere formation (Figure 7A, 7B). Knockdown of JMJD3 abolished the inhibition of Oct4 after administration of paricalcitol (Figure 7C). These results suggest that JMJD3 mediates the inhibitory effect of paricalcitol on the stem cell-like properties in MDA-MB-231 cells.

## DISCUSSION

Accumulating data have implied a positive correlation between stem cell-like characteristics and the malignant potency of breast cancer. As a key property of cancer stem-like cells (CSLCs), tumorigenicity is thought to be one of the most critical indexes for CSLCs in addition to self-renewal and multipotent differentiation [21, 22]. Several epigenetic regulators have been suggested to play important roles in promoting the stemness of CSLCs [23]. Polycomb group protein enhancer of zeste homologue 2 (Ezh2) enhances stem cell-like properties in colon cancer by activation of the p21cip1-Wnt/β-catenin signaling pathway, thus leading to cell cycle arrest at the G1/S phase [24]. JMJD3, identified as a H3K27 demethylase, is also deeply involved in various tumor characteristics, such as proliferation, apoptosis and epithelial–mesenchymal transition (EMT) [25, 26]. However, the role of JMJD3 in the acquirement and maintenance of stemness in tumor is poorly understood. Here, our data demonstrate that ectopic overexpression of JMJD3 suppresses stem cell-like characteristics in breast cancer cells, whereas knockdown of JMJD3 promotes such characteristics.

As epigenetic factor, JMJD3 has been proven to play critical role in the development, stemness maintenance, aging and senescence [27]. Among them, senescence is one of the important mechanisms contributing to the suppression of the uncontrolled proliferation of tumor cells [28]. JMJD3 participates in the regulation of large scale of changes in gene expression associated with cellular senescence such as p16, p21, p53, and RB, which are tumor suppressors control the growth arrest aspect of cellular senescence [7]. Our data also supported that JMJD3 led to the overexpression of p16 in breast cancer cells, dependently of its demethylase activity (data not shown here). However, recent studies proposed another non-demethylase dependent mechanism of JMJD3 via JMJD3-PHF20 axis [11]. In our study, JMJD3 limits the stemness of breast cancer cells manipulating the similar pathway as in the induction of iPSCs. Accordingly, several studies also reported that JMJD3 promotes differentiation and cellular senescence in cancer cells [29, 30]. All these data imply that JMJD3 is involved in the regulation of the reprogramming of cancer cells via diverse molecular mechanisms.

Stemness-related transcription factor Oct4 is not only a key stemness marker but also involved in somatic cell reprogramming *in vitro* [11, 31]. In terms of tumors, high expression of Oct4 was detected in breast cancer stem cells and tumor-initiating cells [16]. In addition, it has been well documented that overexpression of Oct4 leads to tumorigenicity in different types of cancer, separately or together with Sox2 and Nanog [12, 14]. Zhao et al. revealed demethylase-dependent and -independent patterns in the regulatory effect of JMJD3 on iPSCs induction [11]. The latter pattern primarily involves JMJD3 targeting PHF20 for ubiquitination and degradation, therefore hampering the reactivation of Oct4 required for somatic cell reprogramming. Interestingly, our data show that the regulatory effect of JMJD3 on the expression of Oct4 is also independent of its demethylase activity in breast cancer cells. Moreover, Oct4 mediates the suppressive effect of JMJD3 on the stem cell-like characteristics of cancer cells. Similar to the process of iPSCs induction, our study also shows that PHF20 is indispensable for activation of the *OCT4* gene in cancer cells. These results support that dedifferentiation of cells in cancers shares a conserved molecular mechanism with somatic cell reprogramming, even at the level of epigenetic regulation. Direct evidence is still needed to compare the differences and similarities between somatic cell reprogramming and cancer formation.

It has been reported that vitamin D upregulates JMJD3 in colon cancer [20, 32].Furthermore, previous study showed that vitamin D exerted inhibitory effects on Oct4 in breast cancer cells [33, 34]. Therefore, it is reasonable to hypothesize that administration of vitamin D might inhibit tumors by upregulation of JMJD3 and subsequent inhibition of Oct4. Because the major functions of vitamin D, if not all, depend on its receptor, VDR, we used the VDR agonist paricalcitol in this study [19, 35]. Our results revealed that paricalcitol induces upregulation of JMJD3. In addition, administration of paricalcitol suppresses the stem cell-like characteristics and expression of Oct4 in breast cancer cells. Previous studies have shown an inhibitory effect of vitamin D on EMT, another critical pathological event of cancer. The upregulation of JMJD3 by vitamin D ensures precise activation of key genes associated with EMT, including ZEB1, ZEB2, and SNAI1 [20]. Therefore, it suggested that the epigenetic regulator JMJD3 plays a broad role in various aspects of cancer characteristics, and the application of vitamin D might benefit the treatment of cancer at various stages of tumor progression.

## MATERIALS AND METHODS

### Cell lines and reagents

MDA-MB-231 cells were purchased from the Institute for Biological Sciences of the Chinese Academy of Sciences (Shanghai, China) and cultured in L-15 medium (Gibco BRL, Gaithersburg, MD) at 37°C without CO_2_. T47D cells were obtained from the American Type Culture Collection (Manassas, VA) and grown in Dulbecco's modified Eagle's medium (DMEM) in a 5% CO_2_ atmosphere at 37°C. Paricalcitol was purchased from Haoyuan Chemexpress (Shanghai, China).

### Vector construction and establishment of stable cell lines

The human JMJD3 expression plasmid pCMV-HA-JMJD3 was purchased from Addgene (Cambridge MA). To construct a human JMJD3 and Oct4 overexpression vector, human JMJD3 and Oct4 cDNA fragments were cloned into the pLV-EF1α-MCS-IRES-Bsd plasmid (Biosettia, San Diego, CA). The short hairpin RNA (shRNA) sequence for silencing human PHF20 (5′GCAGGAGGAGCTTCGCATATT3′) and human Oct4 (5′GCAACCTGGAGAATTTGTT3′) were designed using RNAi designer on the Invitrogen website. A scrambled sequence was used as a control for the knockdown analysis. The shRNAs were cloned into the pLV-H1-EF1α-puro vector (Biosettia). To establish stable cell lines, MDA-MB-231 and T47D cells were infected with lentiviruses carrying pLV-EF1a-JMJD3-IRES-Bsd (pLV-JMJD3) or the empty vector pLV-EF1α-MCS-IRES-Bsd (pLV-MCS) and then selected by 30 μg/ml blasticidin (Bsd) to generate stable cell lines with JMJD3 overexpression (MDA-MB-231-JMJD3 and T47D-JMJD3) or control cells (MDA-MB-231-MCS and T47D-MCS).

### Transfections

MDA-MB-231, T47D, stable JMJD3-overexpressing or knockdown MDA-MB-231, and control cells were transfected with the pCMV-HA-JMJD3, pCMV vector, pLV-EF1α-Oct4-IRES-Bsd, PHF20 and Oct4 shRNA plasmids, or control vector. A total of 3×10^5^ cells were seeded in a 6-well plate containing medium without penicillin-streptomycin and then transfected using Lipofectamine™ 2000 Transfection Reagent (Invitrogen, Carlsbad, CA) according to the manufacturer's instructions.

### Western blotting

Cells were lysed by radioimmunoprecipitation assay lysis buffer in the presence of protease inhibitor cocktail and phosphatase inhibitor cocktail 2 and 3 (P8340, P5726, and P0044, Sigma-Aldrich). Cell lysates were electrophoresed on 8%–12% SDS-polyacrylamide gels and then transferred to nitrocellulose membranes. Antibodies were against JMJD3, Oct4, UTX and H3K27me3 (ab38113, ab19857, ab36938, and ab6002; Abcam, Inc. Cambridge, MA), aldehyde dehydrogenase (ALDH), β-actin (sc-166362, sc-4778; Santa Cruz Biotechnology, Inc., Santa Cruz, CA) and PHF20 (D96F6, Cell Signaling Technology, Inc., Danvers, MA). The membranes were subsequently incubated with horseradish peroxidase-conjugated secondary antibodies. Bound antibodies were detected by enhanced chemiluminescence reagent (Millipore, Billerica, MA).

### Immunohistochemistry

Paraffin-embedded sections were incubated at 4°C overnight with primary antibodies against Oct4 (Abcam) or ALDH (Santa Cruz Biotechnology) and then incubated with a biotinylated secondary antibody for 90 min, followed by an avidin-peroxidase complex for 30 min. The sections were visualized with diaminobenzidine and counterstained with hematoxylin. The dehydrated sections were mounted with neutral resin.

### RNA isolation, RT-PCR, and real-time PCR

Total RNA was extracted using TRIZOL reagent (Cat#15596, Invitrogen) and reverse transcribed into cDNA. Semi-quantitative RT-PCR was performed by agarose gel electrophoresis. Real-time PCR was performed on a CFXTM Real-Time Thermal cycler (Bio-Rad, Hercules, CA) using a TransStrat Top Green qPCR SupperMix kit (Trans Gen Biotech, Beijing, China). The primers used as follows: homo-JMJD3, sense-5′TGTTCCCTGTAGCACATCAAG 3′ and anti-sense-5′GG TAGAGTGAGTGCGTTTCG3′; homo-UTX, sense-5′CAGAACGGACATCCCACC3′ and anti-sense-5′TCCCATCAACAAGGCAGA3′; homo-Oct4, sense-5′GTATTCAGCCAAA CGACCATC3′ and anti-sense-5′GCTTCCTCCACCCACTTCT3′; homo-PHF20, sense-5′ACCCGGCTCCCCAAAGGTGA3′ and anti-sense-5′CTGCCACTGGTGCTGGGAGC3′; homo-GAPDH, sense-5′CTGATGCCCCCATGTTCGTC3′ and anti-sense-5′CACCCTGTTGCTGATGCCAAATTC3′.

### Tumor initiation analyses

Female BALB/C nude mice aged 6–8 weeks were purchased from Vital River Laboratory Animal Technology Co.Ltd (Beijing, China). The mice were housed at the animal facilities of Nankai University with free access to food and water. All animal experiments were performed strictly under the guidelines on laboratory animals of Nankai University. Mice were separated into two groups (n=5) and subjected to 200 μl (2:1, basic L-15 cells: matrigel, CORNING, New York, NY) subcutaneous injections of breast cancer cells at the second mammary fat pad. Tumorigenesis was assessed every 2 days by manual palpation for 2 weeks following injection. For paricalcitol administration experiments, paricalcitol or the vehicle control were administered via intraperitoneal injection at a dose of 0.3 μg/kg body weight once every other day from 3 days before tumor cell injection. The mice were sacrificed at week 8 after injection, and the tumor tissues were isolated for further analysis.

### Aldefluor assay

An ALDEFLUOR kit (STEMCELL Technologies, Vancouver, Canada) was used to measure ALDH enzymatic activity in breast cancer cells. Briefly, 2.5×10^5^ cells were suspended in ALDRFLUOR assay buffer containing ALDH1 substrate and incubated for 40 min at 37°C. Treatment with DEAB, a specific ALDH inhibitor, served as the negative control. Stained cells were analyzed on BD FACS Calibur flow cytometer (BD Biosciences, San Jose, CA). Data analysis was performed using Flowjo software (Tree Star, Inc., Ashland, OR).

### Sphere formation assay

A total of 1×10^3^ cells per well were seeded in an ultra-low attachment 48-well plate (CORNING). Spheres were grown in serum-free L-15 or DMEM supplemented with 1× B27 (1:50, Invitrogen), 20 ng/ml human epidermal growth factor, and 20 ng/ml basic fibroblast growth factor. For the paricalcitol administration assay, cells were grown in the presence of paricalcitol (1 or 10 nM). Sphere numbers were countered after 7 days.

### Chromatin immunoprecipitation (ChIP)

The CHIP assay was performed using an EZ-ChIP kit (Cat#17-371, Millipore Corp, Billerica, MA) following the manufacturer's instructions. In brief, cells grown in 10cm dishes were cross-linked with 1% formaldehyde for 10 min at room temperature. The reaction was stopped with glycine. Samples were collected, sonicated, and centrifuged, and then the supernatant was collected for immunoprecipitation using an antibody against H3K27me3 (Abcam). Anti-RNA polymerase and normal mouse IgG were used as positive and negative controls, respectively. Semi-quantitative RT-PCR was performed to detect a DNA fragment of the Oct4 promoter. The primers used as follows: CR1, sense-5′GAGGTCAGCGTGCCCAGTCCA3′ and anti-sense-5′CAGGCGTCCAGCTTCATCGTG3′; CR2, sense-5′GGGATGCACGATGAAGCTGGACG3′ and anti-sense-5′CAAAGAAGCCTGGGAGGGACTGG3′; CR3, sense-5′CAGTCCCTCCCAGGCTTCTTT3′ and anti-sense-5′CACATCAGGTTCCTTGCTCCC3′; CR4, sense-5′GGGCCTACCGTGGTATTAGATGT3′ and anti-sense-5′CCTCTCAGCTCCTCAAATTTA3′; CR5, sense-5′GCCTTGTCTGGCAGTCTACTCTT3′ and anti-sense-5′TTTCAACTCCCAACCCGCTCC3′; CR6, sense-5′GGGAGCGGGTTGGGAGTTGAA3′ and anti-sense-5′GGATGTTTGCCTAATGGTGGTG3′; CR7, sense-5′CCACCACCATTAGGCAAACAT3′ and anti-sense-5′CTGGAGGGGGCGAGAAGGCGA3′.

### Dual-luciferase reporter assay

The human Oct4 promoter predicted by software was cloned into the pGL4.2-basic luciferase reporter vector (Promega, Madison, WI) that encodes firefly luciferase (FL). Cells were co-transfected with pCMV-control or pCMV-HA-JMJD3 plasmids. The pRL Renilla luciferase reporter vector (pRL-TK) was used as the internal control. Cell lysates were collected after transfection for 40 h. Oct4 promoter activity was analyzed as the ratio of FL/RL activity following the manufacturer's instructions.

### Statistical analysis

All data were analyzed using GraphPad Prism5 software (GraphPad Software, San Diego, CA). Results are presented as the mean ± standard error of the mean (S.E.M). Statistical significance was determined by the Student's *t*-test or two-way analysis of variance.
